# The LRRK2 signalling system

**DOI:** 10.1007/s00441-017-2759-9

**Published:** 2018-01-08

**Authors:** Alice Price, Claudia Manzoni, Mark R. Cookson, Patrick A. Lewis

**Affiliations:** 1 School of Pharmacy, University of Reading, Whiteknights, Reading, RG6 6AP UK; 2 Department of Molecular Neuroscience, UCL Institute of Neurology, Queen Square, London, WC1N 3BG UK; 3 Laboratory of Neurogenetics, National Institute on Aging, National Institutes of Health, Building. 35, 35 Convent Drive, Bethesda, MD 20892 USA

**Keywords:** LRRK2, Signal transduction, Parkinson’s disease, ROCO protein, Kinase

## Abstract

The *LRRK2* gene is a major contributor to genetic risk for Parkinson’s disease and understanding the biology of the leucine-rich repeat kinase 2 (LRRK2, the protein product of this gene) is an important goal in Parkinson’s research. LRRK2 is a multi-domain, multi-activity enzyme and has been implicated in a wide range of signalling events within the cell. Because of the complexities of the signal transduction pathways in which LRRK2 is involved, it has been challenging to generate a clear idea as to how mutations and disease associated variants in this gene are altered in disease. Understanding the events in which LRRK2 is involved at a systems level is therefore critical to fully understand the biology and pathobiology of this protein and is the subject of this review.

**Electronic supplementary material:**

The online version of this article (10.1007/s00441-017-2759-9) contains supplementary material, which is available to authorized users.

## Introduction

Leucine-rich repeat kinase 2 (LRRK2) is a large (268-kDa) multi-domain protein member of the Roco protein family (Civiero et al. 2014). It is characterised by a complex domain architecture, including two enzyme domains: a ROCO GTPase supradomain incorporating Ras of complex proteins (ROC) and C-terminal of ROC (COR) domains; and a serine/threonine kinase domain. It has been proposed that the kinase activity of LRRK2 is intimately linked to that of the ROC domain. At the N-terminus, protein interaction domains are present including Armadillo, Ankyrin, and leucine-rich repeat domains, whereas the C-terminus includes a WD40 domain. The domain organisation of LRRK2 strongly implicates this protein in the command and control of signal transduction within the cell—borne out by experimental investigations into LRRK2 function, with LRRK2 linked to the regulation of a range of pathways and signal transduction cascades. Many of these are vital for homeostasis, including mechanisms such as proliferation, apoptosis, inflammation, cytokine activation, synaptogenesis and maintenance and autophagy (Wallings et al. 2015).

### LRRK2 in disease

LRRK2 first came to prominence due to the association of the *LRRK2* gene with Parkinson’s disease (PD). In 2002, Funayama et al. described a locus on chromosome 12 linked to PD in a family from the Sagamihara region of Japan (Funayama et al. 2002), and, in 2004, two independent groups identified autosomal dominant mutations in the *LRRK2* gene (Paisán-Ruíz et al. 2004; Zimprich et al. 2004). Subsequent studies have revealed that *LRRK2* is a central player in the genetics of PD, with coding variants being the most common genetic cause of familial disease and more subtle common variation at the *LRRK2* locus linked to genome-wide associated risk (Ross et al. 2011; Trabzuni et al. 2013). One aspect of LRRK2 PD that is in contrast with the idiopathic form of disease is the pathology observed in cases with mutations. Whilst the majority of LRRK2 cases present with the Lewy body pathology associated with classical PD (Hughes et al. 1992), a significant minority present with neurofibrillary tangle pathology similar to that observed in progressive supranuclear palsy or TDP-43 pathology (Wider et al. 2010).

**Fig. 1:**
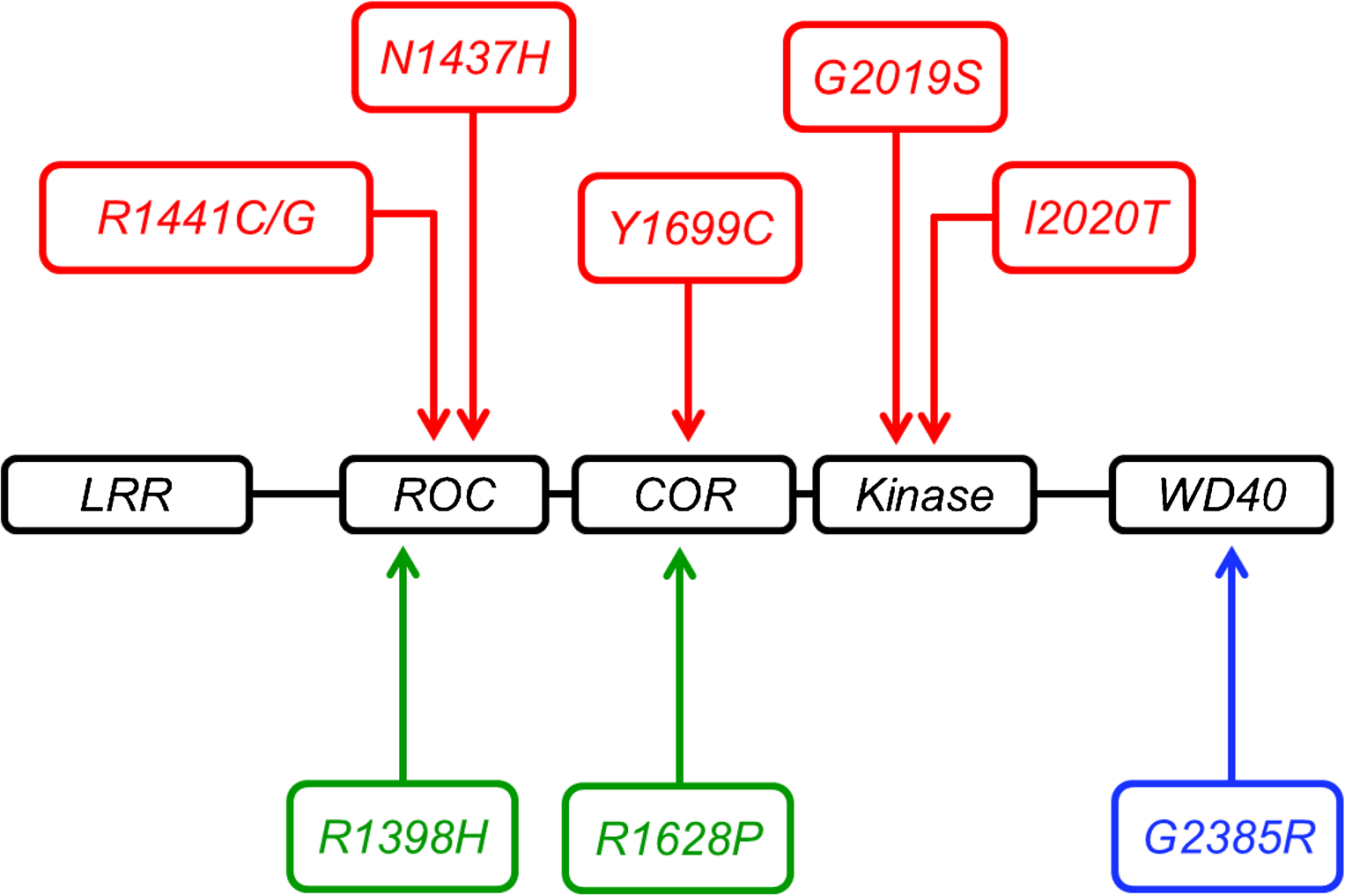
Disease-associated coding variants in LRRK2. Penetrant mutations associated with Parkinson’s disease are displayed in *red*, protective variants in green and disease-associated risk variants in blue

A large number of coding changes in the *LRRK2* gene have been identified but only six have strong evidence for pathogenicity in PD (Ross et al. 2011): R1441C/G, N1437H, Y1699C, G2019S and I2020T (Kalia et al. 2015; Puschmann et al. 2012; Greene et al. 2014). In addition to these highly penetrant causative factors, coding variants in *LRRK2* with increased risk for PD have been identified, such as M1646 T and G2385R, as well as a potentially protective haplotype N551 K-R1398H-K1423 K (Ross et al. 2011) (Fig. 1).

Of the coding variants strongly associated with disease, G2019S is the most prevalent and varies by ethnic background: from 0.1% in Asians and 13% in Ashkenazi Jews to 41% in North African populations (Chien et al. 2014; Li et al. 2014). Interestingly, mutation carriers can be symptom free beyond their 8th decade (Sierra et al. 2011; Trinh et al. 2014). This demonstrates that these mutations show age-dependent but incomplete penetrance. The overall penetrance of G2019S can vary between populations, with a later age of onset and lower penetrance in Norwegian carriers of the G2019S variant compared to other populations (Trinh et al. 2014).

The precise molecular consequences of mutations in LRRK2 are not yet completely clear; however, a common theme is alterations in enzymatic activity. The causative mutations described above all localise to the enzymatic core of LRRK2, spanning the GTPase to Kinase domain and the majority of these coding variants alter LRRK2 enzymatic function. The G2019S mutation has been robustly associated with an increase in kinase activity (Greggio and Cookson 2009), with mutations in the ROC/COR domains (e.g., R1441C, Y1699C) demonstrating reduced GTPase activity (Lewis et al. 2007; Daniẽls et al. 2011). These data, repeatedly highlighting the enzymatic functions of LRRK2 and their role in the control of signalling pathways, strongly suggest that perturbations in signal transduction lie at the heart of LRRK2 dysfunction in PD.

Intriguingly, variation at the *LRRK2* locus has also been connected by genome-wide association with immune diseases such as systemic lupus erythematosus (Zhang et al. 2017), Crohn’s disease (Barrett et al. 2008) and inflammatory bowel disease (Liu et al. 2011) and with susceptibility to multibacillary leprosy (Wang et al. 2015; Fava et al. 2016). LRRK2 has also been linked to cancer, with the PD G2019S mutation associated with specific cancers such as non-skin and hormonal cancers (Agalliu et al. 2015) and the R1441C mutation linked to increased risk of colon cancer (Tacik et al. 2016).

Deciphering what LRRK2 does has, therefore, important ramifications for efforts to target a wide range of human diseases. To achieve this, a key challenge is to develop a detailed understanding of LRRK2’s function as a signalling molecule within the cell, an area of biology that has received an exceptional amount of attention over the past decade.

### LRRK2 as a signalling molecule

As noted above, the specific domains and structure of LRRK2 implicate it in the regulation of cellular signal transduction events, the dissection of which will be critical for understanding LRRK2s cellular function and the role that it plays in disease. This task is somewhat complicated by the extensive list of putative protein binding partners and cellular processes to which LRRK2 has been linked (Manzoni et al. 2015). Determining which of these processes and interactors are physiologically relevant and beyond that relevant to disease, is a significant challenge.

The array of pathways and interactors with which LRRK2 has been linked is at least partly due to the intrinsic scaffolding properties of this protein. Outside of its enzymatic activities, LRRK2 possesses a number of protein/protein interaction motifs. The N-terminus harbours Ankyrin, Armadillo and leucine-rich repeats. Ankyrin repeats are found in molecules with cell–cell signalling functions and typically facilitate protein interactions or assist in protein recognition (Mosavi et al. 2004). Armadillo repeats have been described in structural roles and vesicle dynamics (Striegl et al. 2010). Leucine-rich repeats are often found within proteins with conserved functions in innate immunity involving pathogen recognition (Ng and Xavier 2011). In addition to these domains, a C-terminal WD40 domain is present. This class of domain has been implicated in several functions including protein scaffolding and interactions (Stirnimann et al. 2010). This could indicate repeat regions mediating and supporting kinase function; or a possible role for LRRK2 as a signalling molecule independent of the kinase (Guaitoli et al. 2016).

An added layer of complexity is provided by data demonstrating that LRRK2 can form homodimers, with kinase function reported to be dependant upon this phenomenon (Greggio et al. 2008; Berger et al. 2010). The reciprocal nature of this relationship is evidenced by studies suggesting that disruption of GTPase and kinase activities can act to prevent dimerization, thereby reducing kinase activity. It is of note that some pathogenic mutations cause a shift in dimer formation, producing more dimers compared to single proteins, consequently increasing kinase function whilst decreasing GTPase activity. This demonstrates the complex relationship between LRRK2 structure, dimerization and function, possibly alluding to an intramolecular mechanism to regulate domain function (Sen et al. 2009). In this regard, the absence of an atomic resolution structure for LRRK2 is a major hindrance to detailed mechanistic insight, although this has been partly mitigated by the development of a model for the LRRK2 holoprotein based upon cryo-electron microscopy density mapping (Guaitoli et al. 2016) .

Importantly, the potential link between LRRK2 kinase activity and cellular function, coupled to dysfunction of LRRK2 in disease, specifically the observation of increased kinase activity by some mutations, has spurred the development of small molecule inhibitors targeting this enzymatic activity (Atashrazm and Dzamko 2016). In addition to their potential therapeutic value, these have proven to be powerful tools for investigating the signalling events surrounding LRRK2, for example aiding in the identification and validation of LRRK2 kinase substrates (Steger et al. 2016).

The pathways and signalling events linked to LRRK2 can be broadly divided into those that have evidence of a direct interaction with LRRK2, either through protein/protein interactions or enzymatic activities and those that occur at a distance through multiple intermediaries. Characterising these events in a systematic fashion, and developing a physiologically relevant model for the LRRK2 signalling system, has the potential to be of great use in advancing our understanding of LRRK2’s cellular role.

## Proximal signalling events

A wide range of direct interactions with LRRK2 have been reported in the literature. These relate to phosphorylation events (both of and by LRRK2) and to direct protein/protein interactions.

### Regulation of LRRK2

**Fig. 2:**
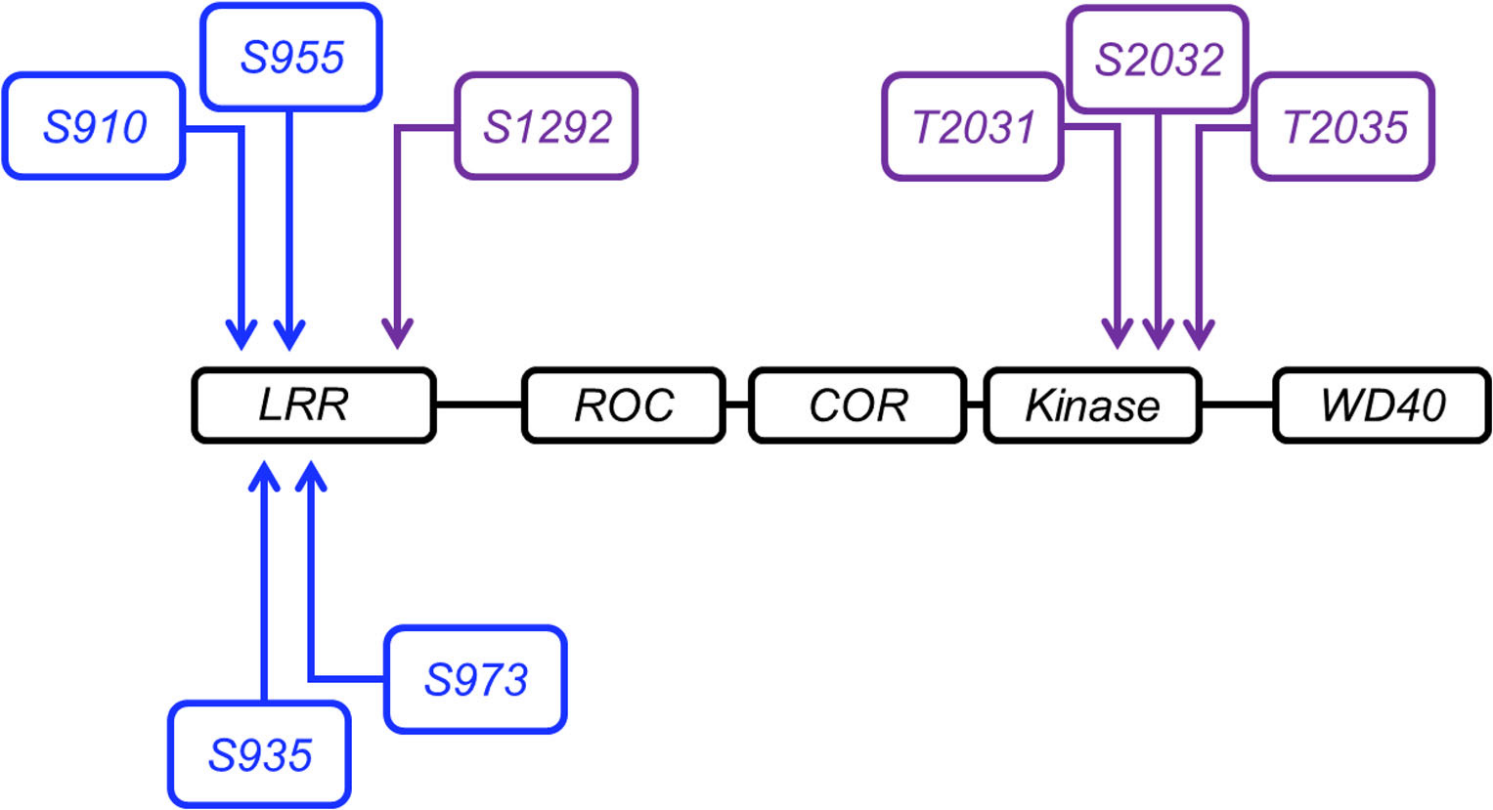
Phospho-events regulating LRRK2. Autophosphorylation is shown in *purple*, phosphorylation events mediated by another kinase in *blue*

As is to be expected of a protein implicated in a range of critical physiological processes, the regulation of LRRK2 described to date is complex, with a number of posttranslational modifications and protein interactions governing the activity of the LRRK2 protein complex. In common with many enzymes involved in signal transduction, phosphorylation appears to play an important role in governing LRRK2 enzymatic activity and function (Fig. 2). Several groups have determined phosphorylation sites on LRRK2 that act to regulate function and downstream signalling events. There is experimental evidence for phosphorylation at S860, S910, S935, S955 and S973 (Doggett et al. 2012); however, not all of these potential sites have been associated with specific kinases or characterised in detail. The precise identity of the kinases involved in these phosphorylation events remains an area of some debate, although there is evidence that the IkappaB kinase family, including tank binding kinase 1 (TBK1), is responsible for a proportion of the phosphorylation found on these residues (Dzamko et al. 2012). Casein kinase I has also been nominated as an upstream regulator of LRRK2 (Chia et al. 2014). LRRK2 has also been shown to undergo autophosphorylation at S1292 (Sheng et al. 2012), T2031, S2032 and T2035 (Li et al. 2010). Phosphorylation at S1292 has an particularly interesting consequence, as this has been shown to regulate the activity of the GTPase domain thereby potentially altering signal transduction, translation and other vital cellular processes (Liu et al. 2016). Protein phosphatase 1 has also been identified as being able to regulate LRRK2 by dephosphorylation (Lobbestael et al. 2013), potentially indicating that further phosphatases could interact in this way.

The phosphorylation of LRRK2 in this region and in particular at S910 and S935, is intimately linked with the ability of LRRK2 to interact with 14–3-3 proteins (Nichols et al. 2010; Li et al. 2011; Doggett et al. 2012). This interaction, in turn, has a key role in cellular localisation of LRRK2 with likely consequences for physiological function. This example clearly demonstrates the deep-seated connection between phospho-regulation, protein/protein interactions and protein function that is central to the control signal transduction by LRRK2—with reciprocal relationships controlling where LRRK2 is located within the cell, how active are its enzymatic activities and with what partners it interacts. Beyond phospho-regulation, LRRK2 activity may be influenced by other posttranslational modifications such as acetylation and glycosylation. There is certainly evidence for regulation of LRRK2 prior to translation via the production of splice isoforms (Trabzuni et al. 2013). These have not yet been subjected to the same level of scrutiny as phosphor-regulation and represent a significant gap in our understanding of LRRK2 biology.

### Regulation of other proteins by LRRK2

As displayed in Table 1, a number of proteins have been reported to be directly regulated by LRRK2, predominantly by phosphorylation. The proportion of these that have been carefully validated, subject to replication by multiple groups and using multiple independent techniques, is, however, much smaller. Examples of direct kinase substrates reported for LRRK2 include a number of members of the mitogen-activated protein kinase family, the microtubule-associated protein Tau, the eukaryotic translation initiation factor 4E–BP1 and several members of the Rab GTPase family. 4E–BP1 provides an interesting case study in the challenges presented by verifying phosphorylation events. This was originally reported as a substrate for LRRK2 in 2008 based upon data in a *Drosophila melanogaster* model for LRRK2 dysfunction (Imai et al. 2008), a study that included in vitro data suggesting that human LRRK2 could directly phosphorylate 4E–BP1. Subsequent studies from a number of groups have failed to conclusively demonstrate that 4E–BP1 acts as a direct substrate for LRRK2, or that 4E–BP1 is dysregulated when LRRK2 activity is altered (Kumar et al. 2010; Trancikova et al. 2012; Manzoni et al. 2013a, b), although there are conflicting data on this (Pons et al. 2012). This example highlights the complexities of validating phosphorylation events, events that can be both transitory and highly context dependent in nature.

**Table 1:** Annotated LRRK2 substrates from protein/protein interaction databases

Name	No. of publications	Direction of interaction
AKT1 PKB RAC	1	A phosphorylates B
ARFGAPI ARF1GAP	2	A phosphorylates B
ARHGEF7 COOL1 KIAA0142 P85SPR PAK3BP PIXB Nbla10314	3	A phosphorylates B
CSNK1A1	1	B phosphorylates A
DNM1L DLP1 DRP1	1	A phosphorylates B
EEF1A2 EEF1AL STN	1	Co-incubation of recombinant LRRK2 and EFTA significantly reduces the kinase activity of LRRK2. Co-incubation of EF1A with LRRK2 impaired tubulin polymerisation by EFTA
ElF4EBP1	2	A phosphorylates B
GSK3B	1	LRRK2 directly associates with GSK-36 and this interaction enhances the kinase activity of GSK-3β
HSPD1 HSP60	1	hsp60 may perform a chaperone action in maintaining the proper folding of recombinant LRRK2 kinase domain in Escherichia coll.
LDHB	1	A phosphorylates B
MAP1B	2	A phosphorylates B
MAP2K3 MEK3 MKK3 PRKMK3 SKK2	2	A phosphorylates B
MAP2K4 JNKK1 MEK4 MKK4 PRKMK4 SEK1 SERK1 SKK1	1	A phosphorylates B
MAP2K6 MEK6 MKK6 PRKMK6 SKK3	2	A phosphorylates B
MAP2K7 JNKK2 MEK7 MKK7 PRKMK7 SKK4	2	A phosphorylates B
MAPT MAPTL MTBT1 TAU	2	A phosphorylates B
MBP	6	A phosphorylates B
MSN	10	A phosphorylates B
PRDX3 AOP1	1	A phosphorylates B
PRKACA PKACA	1	B phosphorylates A
PRKCZ PKC2	1	B phosphorylates A
RAB10	1	A phosphorylates B
RABIA RAB1	1	A phosphorylates B
RAB1B	1	A phosphorylates B
RAB5B	2	A phosphorylates B
RAB8A MEL RAB8	1	A phosphorylates B
RGS2 GOS8 GIG31	1	Recombinant RGS2 inhibited kinase activity of full-length LRRK2 in vitro in a dose-dependent manner
RIPK2 CARDIAK RICK RIP2 UNQ277/PRO314/PRO34092	1	A phosphorylates B
RPL10A NEDD6	1	A phosphorylates B
RPL13 BBC1 OK/SW-c1.46	1	A phosphorylates B
RPL14	1	A phosphorylates B
RPL17	1	A phosphorylates B
RPL21	1	A phosphorylates B
RPL23A	1	A phosphorylates B
RPL30	1	A phosphorylates B
RPL34	1	A phosphorylates B
RPL36A RPL44 GIG15 MIG6	1	A phosphorylates B
RPL39	1	A phosphorylates B
RPS11	1	A phosphorylates B
RPS13	1	A phosphorylates B
RPS15 RIG	2	A phosphorylates B
RPS16	1	A phosphorylates B
RPS18 D6S218E	1	A phosphorylates B
RPS2 RPS4	1	A phosphorylates B
RPS23	1	A phosphorylates B
RPS27 MPS1	1	A phosphorylates B
RPS3 OK/SW-c1.26	1	A phosphorylates B
SH3GL1 CNSA1 SH3D2B	1	A phosphorylates B
SH3GL2 CNSA2 SH3D2A	2	A phosphorylates B
SH3GL3 CNSA3 SH3D2C	1	A phosphorylates B
SNAPIN BLOC1S7 SNAP25BP SNAPAP	1	A phosphorylates B
STK24 MST3 STK3	1	A phosphorylates B
STK25 SOK1 YSK1	1	A phosphorylates B
STK3 KRS1 MST2	1	A phosphorylates B
TAOK3 DPK JIK KDS MAP3K18	1	A phosphorylates B
TP53 P53	1	A phosphorylates B
TUBA1A TUBA3	1	A phosphorylates B
LRRK2 PARKS	26	A phosphorylates A

The terms: APID, Interactomes, BioGrid, bhf-ucl, InnateDB, InnateDB-All, IntAct, mentha, MINT, InnateDB-IMEx, UniProt and MBInfo were searched for LRRK2 interactions and those described as phosphorylation events were extracted. The direction of phosphorylation was determined by manually curating each entry and referring back to the original study. A fully referenced version of this table is available as supplemental Table 1

More recently, a consortium of researchers reported Rab-8 and Rab-10 as phospho-substrates for LRRK2, a study that again provides a valuable example of the challenges in identifying bona fide interactors (Steger et al. 2016). The Rab proteins were identified using a combinatorial proteomic approach, carrying out an unbiased screen of altered phosphorylation in labelled cells following inhibition of LRRK2 kinase activity with a panel of specific inhibitors. These were then validated using a combination of in vitro and cellular approaches, including the generation of phospho-specific antibodies and artificial mutations. These represent probably the most thoroughly validated substrates for LRRK2 to date (other than LRRK2 itself) and yet—as will be discussed further below—despite this the impact of mutations on phosphorylation of these substrates is not yet completely clear. More recent data have highlighted a range of Rab proteins as being potentially phosphorylated by LRRK2, including Rab-3 A/B/C/D, Rab-12, Rab-35 and Rab-43, in addition to Rab-8 and Rab-10 (Steger et al. 2017). Of these, phosphoregulation of Rab-10 and Rab-12 has recently been replicated by an independent group (Thirstrup et al. 2017).

### Distal signalling events

The proximal, immediate signalling events described above have significant consequences downstream in a range of signalling networks and physiological processes. I LRRK2 signalling has been shown in influence autophagy, mitochondrial function, transcription, molecular structural integrity and parts of the immune response. A detailed discussion of the pathways potentially regulated by LRRK2 is beyond the scope of this review; however, there are several excellent recent articles that cover this is area in depth (Wallings et al. 2015; Roosen and Cookson 2016; Kang and Marto 2017).

In 2006, it was found that LRRK2 likely localises to vesicular and membranous structures within neurons—leading to the suggestion that the protein is involved in regulation of vesicular structures within the brain (Biskup et al. 2006). This suggestion has been further investigated by numerous groups and has expanded to encompass synaptic vesicle trafficking (Cirnaru et al. 2014), endosomal and trans-Golgi sorting (MacLeod et al. 2013; Beilina et al. 2014) and autophagy (Plowey et al. 2008; Alegre-Abarrategui et al. 2009). Of these, autophagy is one of the most intensively studied; however, many aspects of this are still not fully understood. A number of these studies have focused on the expression of G2019S LRRK2 and indicate that this mutation promotes an increase of autophagic vacuoles and other autophagic abnormalities in both SH-SY5Y cells (Plowey et al. 2008) and transgenic mice (Ramonet et al. 2011).

Several studies have suggested that mitochondrial dysfunction could be mediated by LRRK2 in some way. Some PD-associated mutations, such as G2019S, exacerbate the wild-type LRRK2 function of mitochondrial fragmentation causing release of cytotoxic and damaging substances (Wang et al. 2012). These damaged organelles would be subject to degradation by autophagy (specifically via mitophagy, a specialised form of autophagy)—another function that has been shown to be influenced by LRRK2.

Another process that has been linked to LRRK2 is transcriptional regulation, as reviewed in detail by Berwick and Harvey (2011). Further to this, groups have altered LRRK2 expression to identify specific functions and molecules impacted by a modulation of transcription. Nikonova et al. (2012) compared gene expression in transgenic LRRK2 wild-type, knockout and G2019S mutated mice. It was found that transcription activity was upregulated in G2019S mice compared to knockout and wild-type when each LRRK2 condition was overexpressed. This suggests that LRRK2 has some function or influence on the transcription process (Nikonova et al. 2012), although again the data on this are conflicting (Devine et al. 2011).

It has been shown that LRRK2 is abundantly expressed in immune cells and enhances NF-κB-dependent transcription, thereby suggesting a role within immune signalling. A number of studies have revealed a deficiency or alteration in the immune response upon knockdown of LRRK2 (Kim et al. 2012; Wandu et al. 2015) and, additionally, LRRK2 has been shown to be a potential substrate for proteins such as the cytokine IFN-γ (Gardet et al. 2010) and the IKK family of kinases, including TBK1 (Dzamko et al. 2012). Whilst this does not conclusively associate LRRK2 with immune function at a molecular level, there is clearly a potential influence that merits further investigation, especially given genetic evidence linking LRRK2 to disorders with a strong immune component.

### Mutations in the context of the LRRK2 signalling system

As noted above, one of the key drivers of research into LRRK2 is the important role that this protein plays in the etiogenesis of PD. Given the clear impact of mutations in LRRK2 enzymatic activities of this protein, it follows that these mutations will have a significant impact on the signalling events regulated by LRRK2. Discerning a clear pattern in precisely how, and in what direction, mutations perturb LRRK2-dependent signalling has, however, proved a significant challenge. Based on data from purified recombinant protein, there is not a clear consistent impact of LRRK2 mutations on intrinsic kinase or GTPase activity. The G2019S variant results in heightened kinase activity as measured by a wide range of putative substrates but this is not matched in vitro by other disease-associated variants (Greggio and Cookson 2009). There is a consistent decrease in intrinsic GTP hydrolysis in the presence of mutations in the ROC and COR domains (e.g., the R1441C and Y1699C mutations); however, this activity has not been exhaustively examined for disease-associated variants in the kinase domain such as G2019S and there is some evidence that GTP hydrolysis is increased by the I2020T variant (Lewis et al. 2007; Xiong et al. 2010; Daniẽls et al. 2011; Tsika and Moore 2013; Ho et al. 2016).

To further confuse matters, when downstream phosphorylation events are examined in cellular systems, disease-associated variants appear to do different things depending on the specific event. This can be illustrated by examining three examples: phosphorylation of LRRK2 on S910/S935 (phosphorylation events that are not due to autophosphorylation but display some dependency on LRRK2 kinase activity), phosphorylation at S1292 (thought to be autophosphorylation) and phosphorylation of Rab-8 and Rab-10. In the case of the former, phosphorylation at S910 and S935 is significantly decreased by the R1441G, Y1699C and I2020T mutations but not altered by the R1441C and G2019S mutations (Nichols et al. 2010; Li et al. 2011). Notably, kinase-dead forms of LRRK2 do not altered S910/S935 phosphorylation, although acute inhibition of kinase activity does decrease phosphorylation at these residues (Dzamko et al. 2010). Phosphorylation at S1292 is increased by PD-associated mutations in LRRK2, with the exception of Y1699C, and is dependent upon LRRK2 kinase and GTPase activity (Sheng et al. 2012; Steger et al. 2016). Phosphorylation of Rab-8 and Rab-10 at T72 and T73, respectively, is increased by mutations in LRRK2, other than the G2019S variant, when examined in cells (Steger et al. 2016). Examined in vitro, however, the G2019S increases phosphorylation of Rab-8 at T72 and the R1441C variant is indistinguishable from wild-type, the opposite of what is observed in cells. These data are summarised in Table 2. It is also noteworthy that the mutation of S910 and S935 to alanine residues does not alter phosphorylation of S1292 (thought to be an autophosporylation event) but does alter phosphorylation of Rab-10 (Ito et al. 2016).

**Table 2:**
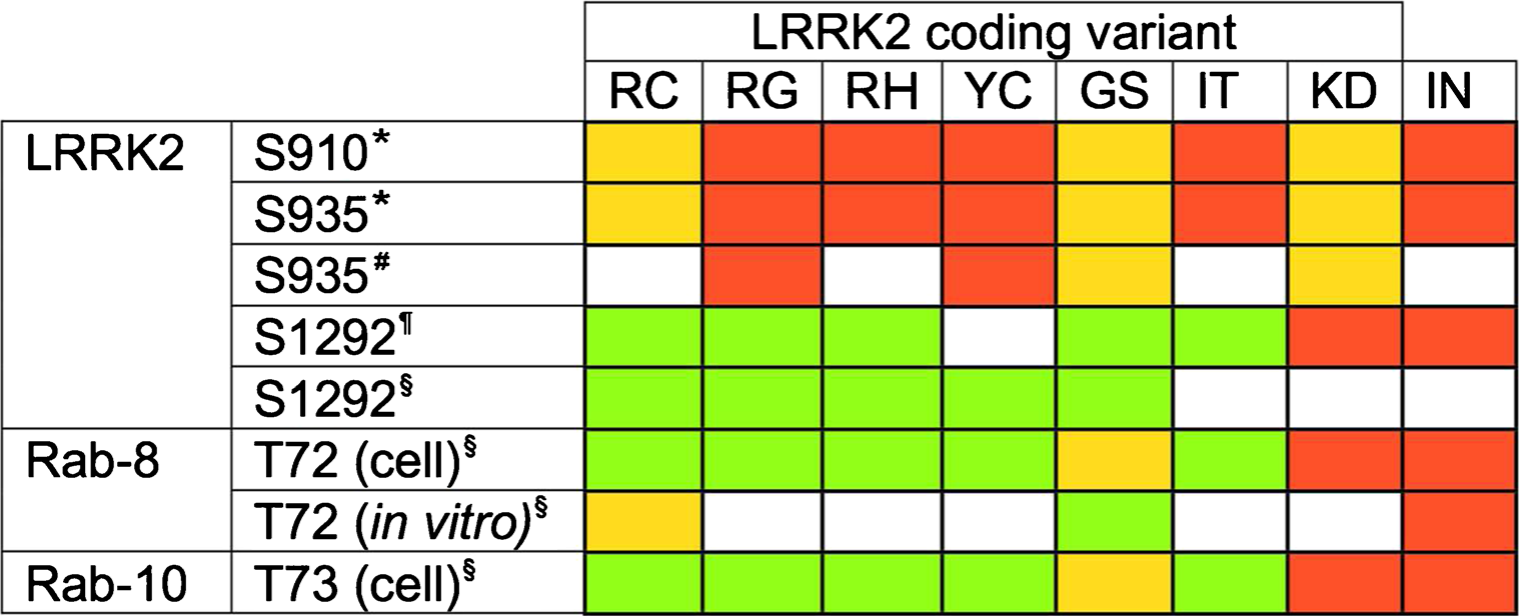
Impact of mutations and inhibition on phosphorylation events associated with LRRK2. Increased phosphorylation is indicated in green, decreased phosophorylation in red, no significant chagne in yellow and no date in white

Mutations: R1441C (RC), R1441G (RG), R1441H (RH), Y1699C (YC), G2019S (GS), I2020T (IT), Kinase dead (KD), inhibitor treatment (IN). Data from indicated references: * Nichols et al. 2010, Dzamko et al. 2010 ^#^ Li et al. 2011 ^¶^ Sheng et al. 2012 ^**§**^ Steger et al. 2016

A reasonable interpretation of the available data is that the in vitro data do not fully recapitulate the activity of LRRK2 in intact systems. It is likely that LRRK2-dependent phosphorylation events are influenced by GTP–GDP turnover in cells, which might in turn be related to membrane association and/or dimerization of LRRK2. Localisation and allosteric changes in the structure of the LRRK2 complex, as well as the presence or absence of co-factors (protein or otherwise), could also underly the discrepancies between in vitro and ex vivo*/*in vivo data. Disentangling these possibilities presents a major challenge to the field but will be important for resolving some of the inconsistencies in the literature between data from recombinant protein and those in cells.

### Modelling the LRRK2 signalling system

**Fig. 3:**
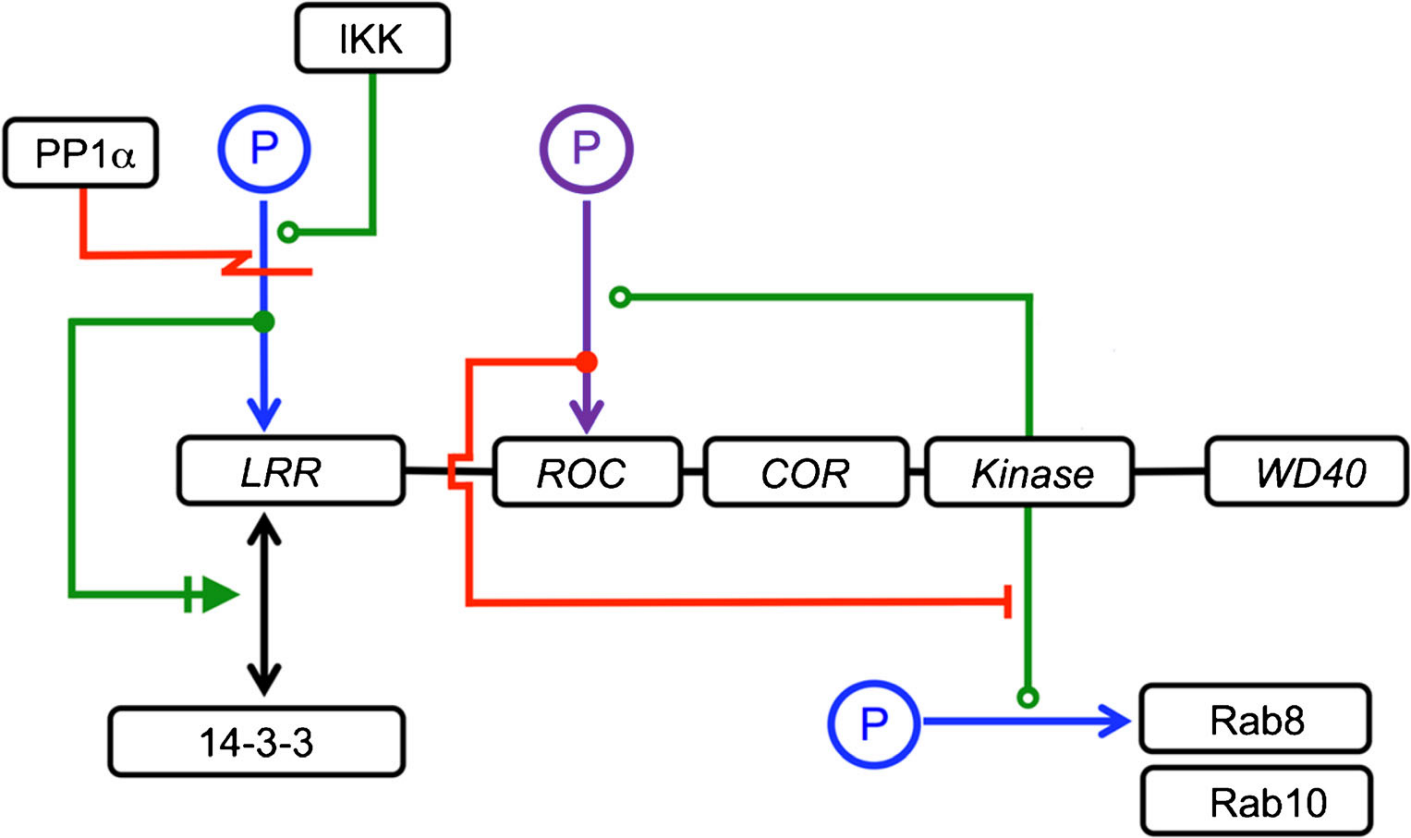
An example of a simplified regulatory network for LRRK2 signalling biology, showing phosphorylation events and protein/protein interactions

Delineating the consequences of mutations in LRRK2 and the impact of manipulating this enzyme through the use of small-molecule inhibitors relies on a high-resolution map of the signalling events surrounding this protein. Although many of the details of LRRK2-dependent signalling remain to be clarified, significant progress has been made in recent years both at a structural and a cellular level (Guaitoli et al. 2016; Steger et al. 2016). Bringing these data together into a comprehensive template for LRRK2 signalling has many potential benefits for research into LRRK2. First, it can provide a framework for developing and testing hypotheses centred around LRRK2 function in signalling networks. This approach has proved extremely valuable in analysing other complex signalling events, for example the regulation and activity of the mammalian/mechanistic target of the rapamycin (mTOR) kinase complex (Caron et al. 2010). It should be noted, however, that the level of system resolution for mTOR signalling—including detailed, verified interaction mapping and Ångström resolution structures—is much higher than our current comprehension of LRRK2 signalling (Kang et al. 2013; Aylett et al. 2016). The power provided by systems level network models is exemplified by the application of this to macroautophagy downstream of mTOR (pertinent to studies of LRRK2 given the reported links between LRRK2 and autophagy), allowing an integrated overview of what is an extremely complex and dynamic process (Kramer et al. 2017). At an individual signalling event level, developing a system map for LRRK2 opens the possibility of constructing mathematical models for regulation of and by LRRK2 based upon detailed time course datasets for LRRK2-mediated signalling events. Again, similar approaches applied to other signal transduction pathways have proved very valuable, for example yielding insights into GPVI signalling (Dunster et al. 2015). An example of how a systems approach could be applied to LRRK2 regulation is provided in Fig. 3, focusing on a group of signalling events that have been characterised in detail: phosphoregulation of S910 and S935, 14–3-3 binding and phosphorylation of Rab 8 and 10. The network is annotated using an established rubric for bioregulatory networks (Kohn et al. 2005). These individual events are suitable for detailed mechanistic investigation, generating datasets that can be used to elucidate kinetic parameters, which, in turn, can be used to generate mathematical models describing these events. Once integrated, these can then be used to generate predictions as to how the system will react to perturbation with, for example, LRRK2 kinase inhibitors, or by mutations with known (and quantified) impacts on kinase/GTPase activity. Any observed differences between these predictions and experimentally generated data from cellular models can then be fed into an iterative process whereby mathematical models for LRRK2 function can be modified to mirror experimental data.

## Conclusions

LRRK2 has been implicated in many functions, both directly through protein interaction and phosphorylation and indirectly through signal transduction cascades. Gaining a deeper understanding of these pathways is critical for comprehending LRRK2s physiological function as well as its role in the multitude of diseases to which it has been linked. As outlined, thinking about LRRK2 as part of a signalling system provides a potentially valuable tool to tackle this challenge. This will be of particular importance as LRRK2 inhibitors edge closer to clinical trials, especially as there is clear evidence of on-target alterations in key organs including the lung (Fuji et al. 2015). In particular, the potential for downstream LRRK2-specific phosphorylation events to act as biomarkers for disease or for evidence of target engagement may have a critical role in assessing LRRK2 kinase inhibitors in first-in-human trials (Fan et al. 2017; Lis et al. 2017). Looking beyond disease, the multi-activity nature of the LRRK2 protein complex has the potential to yield important insights into the fundamental rules governing signal transduction, ensuring that this fascinating protein will remain the subject of detailed investigation for the foreseeable future.

## Electronic supplementary material

ESM 1 (DOCX 21 kb)

ESM 2 A full version of Table 1 including references is available as supplemental Table 1. (XLSX 78 kb)
