# Supplementary material for: The LRRK2 signalling system
Source: Cell Tissue Res. 2018 Jan 8;373(1):39–50. doi: 10.1007/s00441-017-2759-9 (PMC6015615; doi:10.1007/s00441-017-2759-9)
Supplement: Supplementary file 1 — (DOCX 21 kb) [file 441_2017_2759_MOESM1_ESM.docx]

# The LRRK2 Signalling System

Alice Price, Claudia Manzoni, Mark R. Cookson and Patrick A. Lewis

**Supplemental references for supplemental table 1**

Angeles DC, Gan B-H, Onstead L, et al (2011) Mutations in LRRK2 increase phosphorylation of peroxiredoxin 3 exacerbating oxidative stress-induced neuronal death. Hum Mutat 32:1390–7. doi: 10.1002/humu.21582

Arranz AM, Delbroek L, Van Kolen K, et al (2015) LRRK2 functions in synaptic vesicle endocytosis through a kinase-dependent mechanism. J Cell Sci 128:541–52. doi: 10.1242/jcs.158196

Bailey RM, Covy JP, Melrose HL, et al (2013) LRRK2 phosphorylates novel tau epitopes and promotes tauopathy. Acta Neuropathol 126:809–827. doi: 10.1007/s00401-013-1188-4

Biosa A, Trancikova A, Civiero L, et al (2013) GTPase activity regulates kinase activity and cellular phenotypes of Parkinson’s disease-associated LRRK2. Hum Mol Genet 22:1140–56. doi: 10.1093/hmg/dds522

Chan SL, Chua L-L, Angeles DC, Tan E-K (2014) MAP1B rescues LRRK2 mutant-mediated cytotoxicity. Mol Brain 7:29. doi: 10.1186/1756-6606-7-29

Chia R, Haddock S, Beilina A, et al (2014) Phosphorylation of LRRK2 by casein kinase 1α regulates trans-Golgi clustering via differential interaction with ARHGEF7. Nat Commun 5:5827. doi: 10.1038/ncomms6827

Cirnaru MD, Marte A, Belluzzi E, et al (2014) LRRK2 kinase activity regulates synaptic vesicle trafficking and neurotransmitter release through modulation of LRRK2 macro-molecular complex. Front Mol Neurosci 7:49. doi: 10.3389/fnmol.2014.00049

Civiero L, Vancraenenbroeck R, Belluzzi E, et al (2012) Biochemical characterization of highly purified leucine-rich repeat kinases 1 and 2 demonstrates formation of homodimers. PLoS One 7:e43472. doi: 10.1371/journal.pone.0043472

Deng J, Lewis PA, Greggio E, et al (2008) Structure of the ROC domain from the Parkinson’s disease-associated leucine-rich repeat kinase 2 reveals a dimeric GTPase. Proc Natl Acad Sci U S A 105:1499–504. doi: 10.1073/pnas.0709098105

Dusonchet J, Li H, Guillily M, et al (2014) A Parkinson’s disease gene regulatory network identifies the signaling protein RGS2 as a modulator of LRRK2 activity and neuronal toxicity. Hum Mol Genet 23:4887–905. doi: 10.1093/hmg/ddu202

Gillardon F (2009a) Interaction of elongation factor 1-alpha with leucine-rich repeat kinase 2 impairs kinase activity and microtubule bundling in vitro. Neuroscience 163:533–9. doi: 10.1016/j.neuroscience.2009.06.051

Gillardon F (2009b) Leucine-rich repeat kinase 2 phosphorylates brain tubulin-beta isoforms and modulates microtubule stability--a point of convergence in parkinsonian neurodegeneration? J Neurochem 110:1514–22. doi: 10.1111/j.1471-4159.2009.06235.x

Gloeckner CJ, Kinkl N, Schumacher A, et al (2006) The Parkinson disease causing LRRK2 mutation I2020T is associated with increased kinase activity. Hum Mol Genet 15:223–32. doi: 10.1093/hmg/ddi439

Gloeckner CJ, Schumacher A, Boldt K, Ueffing M (2009) The Parkinson disease-associated protein kinase LRRK2 exhibits MAPKKK activity and phosphorylates MKK3/6 and MKK4/7, in vitro. J Neurochem 109:959–68. doi: 10.1111/j.1471-4159.2009.06024.x

Greggio E, Taymans J-M, Zhen EY, et al (2009) The Parkinson’s disease kinase LRRK2 autophosphorylates its GTPase domain at multiple sites. Biochem Biophys Res Commun 389:449–54. doi: 10.1016/j.bbrc.2009.08.163

Greggio E, Zambrano I, Kaganovich A, et al (2008) The Parkinson disease-associated leucine-rich repeat kinase 2 (LRRK2) is a dimer that undergoes intramolecular autophosphorylation. J Biol Chem 283:16906–16914. doi: 10.1074/jbc.M708718200

Guaitoli G, Raimondi F, Gilsbach BK, et al (2016) Structural model of the dimeric Parkinson’s protein LRRK2 reveals a compact architecture involving distant interdomain contacts. Proc Natl Acad Sci 113:E4357–E4366. doi: 10.1073/pnas.1523708113

Guo L, Gandhi PN, Wang W, et al (2007) The Parkinson’s disease-associated protein, leucine-rich repeat kinase 2 (LRRK2), is an authentic GTPase that stimulates kinase activity. Exp Cell Res 313:3658–70. doi: 10.1016/j.yexcr.2007.07.007

Haebig K, Gloeckner CJ, Miralles MG, et al (2010) ARHGEF7 (Beta-PIX) acts as guanine nucleotide exchange factor for leucine-rich repeat kinase 2. PLoS One 5:e13762. doi: 10.1371/journal.pone.0013762

Ho DH, Jang J, Joe E-H, et al (2016) G2385R and I2020T Mutations Increase LRRK2 GTPase Activity. Biomed Res Int 2016:7917128. doi: 10.1155/2016/7917128

Ho DH, Kim H, Kim J, et al (2015) Leucine-Rich Repeat Kinase 2 (LRRK2) phosphorylates p53 and induces p21WAF1/CIP1 expression. Mol Brain 8:54. doi: 10.1186/s13041-015-0145-7

Hsu CH, Chan D, Greggio E, et al (2010) MKK6 binds and regulates expression of Parkinson’s disease-related protein LRRK2. J Neurochem 112:1593–604. doi: 10.1111/j.1471-4159.2010.06568.x

Imai Y, Gehrke S, Wang H-Q, et al (2008) Phosphorylation of 4E-BP by LRRK2 affects the maintenance of dopaminergic neurons in Drosophila. EMBO J 27:2432–43. doi: 10.1038/emboj.2008.163

Ito G, Okai T, Fujino G, et al (2007) GTP binding is essential to the protein kinase activity of LRRK2, a causative gene product for familial Parkinson’s disease. Biochemistry 46:1380–8. doi: 10.1021/bi061960m

Jaleel M, Nichols RJ, Deak M, et al (2007) LRRK2 phosphorylates moesin at threonine-558: characterization of how Parkinson’s disease mutants affect kinase activity. Biochem J 405:307–317. doi: 10.1042/BJ20070209

Jorgensen ND, Peng Y, Ho CC-Y, et al (2009) The WD40 domain is required for LRRK2 neurotoxicity. PLoS One 4:e8463. doi: 10.1371/journal.pone.0008463

Kawakami F, Shimada N, Ohta E, et al (2014) Leucine-rich repeat kinase 2 regulates tau phosphorylation through direct activation of glycogen synthase kinase-3β. FEBS J 281:3–13. doi: 10.1111/febs.12579

Kawakami F, Yabata T, Ohta E, et al (2012) LRRK2 phosphorylates tubulin-associated tau but not the free molecule: LRRK2-mediated regulation of the tau-tubulin association and neurite outgrowth. PLoS One 7:e30834. doi: 10.1371/journal.pone.0030834

Klein CL, Rovelli G, Springer W, et al (2009) Homo- and heterodimerization of ROCO kinases: LRRK2 kinase inhibition by the LRRK2 ROCO fragment. J Neurochem 111:703–15. doi: 10.1111/j.1471-4159.2009.06358.x

Kumar A, Greggio E, Beilina A, et al (2010) The Parkinson’s disease associated LRRK2 exhibits weaker in vitro phosphorylation of 4E-BP compared to autophosphorylation. PLoS One 5:e8730. doi: 10.1371/journal.pone.0008730

Lee S, Liu H-P, Lin W-Y, et al (2010) LRRK2 kinase regulates synaptic morphology through distinct substrates at the presynaptic and postsynaptic compartments of the Drosophila neuromuscular junction. J Neurosci 30:16959–69. doi: 10.1523/JNEUROSCI.1807-10.2010

Li X, Moore DJ, Xiong Y, et al (2010) Reevaluation of phosphorylation sites in the parkinson disease-associated leucine-rich repeat kinase 2. J Biol Chem 285:29569–29576. doi: 10.1074/jbc.M110.127639

Luzón-Toro B, de la Torre ER, Delgado A, et al (2007) Mechanistic insight into the dominant mode of the Parkinson’s disease-associated G2019S LRRK2 mutation. Hum Mol Genet 16:2031–2039. doi: 10.1093/hmg/ddm151

Martin I, Kim JW, Lee BD, et al (2014) Ribosomal protein s15 phosphorylation mediates LRRK2 neurodegeneration in Parkinson’s disease. Cell 157:472–485. doi: 10.1016/j.cell.2014.01.064

Matta S, Van Kolen K, da Cunha R, et al (2012) LRRK2 controls an EndoA phosphorylation cycle in synaptic endocytosis. Neuron 75:1008–21. doi: 10.1016/j.neuron.2012.08.022

Muda K, Bertinetti D, Gesellchen F, et al (2014) Parkinson-related LRRK2 mutation R1441C/G/H impairs PKA phosphorylation of LRRK2 and disrupts its interaction with 14-3-3. Proc Natl Acad Sci U S A 111:E34-43. doi: 10.1073/pnas.1312701111

Nichols RJ, Dzamko N, Morrice NA, et al (2010) 14-3-3 binding to LRRK2 is disrupted by multiple Parkinson’s disease-associated mutations and regulates cytoplasmic localization. Biochem J 430:393–404. doi: 10.1042/BJ20100483

Ohta E, Kawakami F, Kubo M, Obata F (2011) LRRK2 directly phosphorylates Akt1 as a possible physiological substrate: Impairment of the kinase activity by Parkinson’s disease-associated mutations. FEBS Lett 585:2165–2170. doi: 10.1016/j.febslet.2011.05.044

Ray S, Bender S, Kang S, et al (2014) The Parkinson disease-linked LRRK2 protein mutation I2020T stabilizes an active state conformation leading to increased kinase activity. J Biol Chem 289:13042–53. doi: 10.1074/jbc.M113.537811

Reynolds A, Doggett EA, Riddle SM, et al (2014) LRRK2 kinase activity and biology are not uniformly predicted by its autophosphorylation and cellular phosphorylation site status. Front Mol Neurosci 7:54. doi: 10.3389/fnmol.2014.00054

Rudenko IN, Kaganovich A, Hauser DN, et al (2012) The G2385R variant of leucine-rich repeat kinase 2 associated with Parkinson’s disease is a partial loss-of-function mutation. Biochem J 446:99–111. doi: 10.1042/BJ20120637

Sen S, Webber PJ, West AB (2009) Dependence of leucine-rich repeat kinase 2 (LRRK2) kinase activity on dimerization. J Biol Chem 284:36346–36356. doi: 10.1074/jbc.M109.025437

Stafa K, Trancikova A, Webber PJ, et al (2012) GTPase activity and neuronal toxicity of Parkinson’s disease-associated LRRK2 is regulated by ArfGAP1. PLoS Genet 8:e1002526. doi: 10.1371/journal.pgen.1002526

Steger M, Tonelli F, Ito G, et al (2016) Phosphoproteomics reveals that Parkinson’s disease kinase LRRK2 regulates a subset of Rab GTPases. Elife. doi: 10.7554/eLife.12813

Su Y-C, Qi X (2013) Inhibition of excessive mitochondrial fission reduced aberrant autophagy and neuronal damage caused by LRRK2 G2019S mutation. Hum Mol Genet 22:4545–61. doi: 10.1093/hmg/ddt301

Webber PJ, Smith AD, Sen S, et al (2011) Autophosphorylation in the leucine-rich repeat kinase 2 (LRRK2) GTPase domain modifies kinase and GTP-binding activities. J Mol Biol 412:94–110. doi: 10.1016/j.jmb.2011.07.033

West AB, Moore DJ, Biskup S, et al (2005) Parkinson’s disease-associated mutations in leucine-rich repeat kinase 2 augment kinase activity. Proc Natl Acad Sci U S A 102:16842–7. doi: 10.1073/pnas.0507360102

Xiong Y, Yuan C, Chen R, et al (2012) ArfGAP1 is a GTPase activating protein for LRRK2: reciprocal regulation of ArfGAP1 by LRRK2. J Neurosci 32:3877–86. doi: 10.1523/JNEUROSCI.4566-11.2012

Yun HJ, Kim H, Ga I, et al (2015) An early endosome regulator, Rab5b, is an LRRK2 kinase substrate. J Biochem 157:485–95. doi: 10.1093/jb/mvv005

Yun HJ, Park J, Ho DH, et al (2013) LRRK2 phosphorylates Snapin and inhibits interaction of Snapin with SNAP-25. Exp Mol Med 45:e36. doi: 10.1038/emm.2013.68

Zach S, Felk S, Gillardon F (2010) Signal transduction protein array analysis links LRRK2 to Ste20 kinases and PKC zeta that modulate neuronal plasticity. PLoS One 5:e13191. doi: 10.1371/journal.pone.0013191
